# Transcriptional regulators of Na,K-ATPase subunits

**DOI:** 10.3389/fcell.2015.00066

**Published:** 2015-10-26

**Authors:** Zhiqin Li, Sigrid A. Langhans

**Affiliations:** Nemours Center for Childhood Cancer Research, Nemours/Alfred I. duPont Hospital for ChildrenWilmington, DE, USA

**Keywords:** Na,K-ATPase α-subunit, Na,K-ATPase β-subunit, transcription, promoter analysis, cancer, epigenetics

## Abstract

The Na,K-ATPase classically serves as an ion pump creating an electrochemical gradient across the plasma membrane that is essential for transepithelial transport, nutrient uptake and membrane potential. In addition, Na,K-ATPase also functions as a receptor, a signal transducer and a cell adhesion molecule. With such diverse roles, it is understandable that the Na,K-ATPase subunits, the catalytic α-subunit, the β-subunit and the FXYD proteins, are controlled extensively during development and to accommodate physiological needs. The spatial and temporal expression of Na,K-ATPase is partially regulated at the transcriptional level. Numerous transcription factors, hormones, growth factors, lipids, and extracellular stimuli modulate the transcription of the Na,K-ATPase subunits. Moreover, epigenetic mechanisms also contribute to the regulation of Na,K-ATPase expression. With the ever growing knowledge about diseases associated with the malfunction of Na,K-ATPase, this review aims at summarizing the best-characterized transcription regulators that modulate Na,K-ATPase subunit levels. As abnormal expression of Na,K-ATPase subunits has been observed in many carcinoma, we will also discuss transcription factors that are associated with epithelial-mesenchymal transition, a crucial step in the progression of many tumors to malignant disease.

## Introduction

Na,K-ATPase is an integral membrane protein that mainly functions as an ion pump, hydrolyzing one molecule of ATP to pump three Na^+^ out of the cell in exchange for two K^+^ entering the cell per pump cycle. This function is crucial to maintaining the ion gradient across the membrane and is critical for the resting membrane potential, electrical activity of muscle and nerve, Na^+^-coupled transport, and osmotic balance and cell volume regulation (Blanco et al., 1998; Kaplan, 2002). Besides being an ion pump, Na,K-ATPase acts as a signal transducer (Xie and Askari, 2002; Pierre and Xie, 2006; Rajasekaran and Rajasekaran, 2009; Reinhard et al., 2013). Both α- and β-subunit associate with various signaling molecules, including Src, phosphoinositide 3-kinase (PI3K), caveolin-1, protein phosphatase 2, and EGFR thereby activating a number of intracellular signaling pathways, including MAPK and Akt signaling, to modulate cell polarity, cell growth, cell motility and gene expression (Haas et al., 2002; Barwe et al., 2005; Cai et al., 2008; Kimura et al., 2011). Na,K-ATPase also functions as a receptor for the cardiac glycoside ouabain, a specific inhibitor of the pump. Studies with ouabain at concentrations that do not inhibit the Na,K-ATPase pump function have been particularly useful in elucidating the pump-independent signaling pathways associated with Na,K-ATPase (Xie and Askari, 2002; Silva and Soares-da-Silva, 2012; Reinhard et al., 2013). Na,K-ATPase also regulates the formation and stabilization of intercellular junctions and the β_1_ and β_2_-subunits act as cell adhesion molecules (Gloor et al., 1990; Rajasekaran et al., 2001a,b; Vagin et al., 2006, 2012).

Na,K-ATPase is an abundantly expressed protein and can use up to about 2/3 of the energy expenditure of a cell (Howarth et al., 2012) thus requiring tight regulation. Aberrant expression and/or function of Na,K-ATPase has been associated with many disorders, with cancer being one of the more recent ones (Litan and Langhans, 2015). This review will focus on the transcriptional regulation of Na,K-ATPase by hormones, transcription factors, growth factors, extracellular stimuli, and epigenetic modification in general; followed by the role of transcription factors involved in epithelial-mesenchymal transition (EMT) and carcinoma.

## The expression profile of Na,K-ATPase

Na,K-ATPase is a heteromeric transmembrane protein composed of an essential α- and β-subunit (Lingrel and Kuntzweiler, 1994), and an optional third subunit belonging to the FXYD proteins which are more tissue specific regulatory subunits of the enzyme (Geering, 2006). The α-subunit is the catalytic subunit responsible for transport activities of the enzyme. It contains 10 transmembrane segments; a large intracellular domain and a simple extracellular domain. An ATP-binding site and a phosphorylation site locate within the large cytoplasmic loops. The extracellular domain and the transmembrane regions harbor the binding sites for cardiac glycosides such as digoxin and ouabain (Kaplan, 2002; Sandtner et al., 2011; Laursen et al., 2013). The β-subunit is a highly glycosylated transmembrane protein, which increases the translation efficiency and stability of the α-subunit and targets the α-subunit to the plasma membrane (Blanco et al., 1998; Rajasekaran et al., 2004; Benarroch, 2011). It also modulates the affinity of Na^+^ and K^+^ to the enzyme (Blanco et al., 1998) and functions as a cell adhesion molecule (Gloor et al., 1990; Rajasekaran et al., 2001b; Barwe et al., 2005; Kitamura et al., 2005; Shoshani et al., 2005; Vagin et al., 2006, 2012). Unlike the α-subunit, the β-subunit spans the plasma membrane only once and has a very short intracellular N-terminus and a large extracellular C-terminal domain that contains several N-glycosylation sites (Blanco et al., 1998; Benarroch, 2011). In recent years, the FXYD family has been identified as another subunit of Na,K-ATPase. They are a group of small, single-span membrane proteins that are characterized by the FXYD motif in the extracellular domain, two conserved glycine residues within the membrane, and a serine residue at the membrane-cytoplasm interface (Garty and Karlish, 2006; Geering, 2008). FXYD proteins modify the affinity for Na^+^, K^+^, and ATP, pump kinetics and transport properties and stabilize Na,K-ATPase (Garty and Karlish, 2006; Geering, 2006, 2008; Mishra et al., 2011).

The Na,K-ATPase subunits are highly conserved across species and among isoforms. Four isoforms of α-subunit (α_1_–α_4_) and three isoforms of β-subunit (β_1_–β_3_) have been identified so far, which are encoded by different genes (Blanco, 2005). In human, there is high identity in amino acid sequence among the α-subunit isoforms with 87% identity between α_2_ and α_1_ and 88% between α_3_ and α_1_. α_4_ is a little more divergent, but still sharing 79% identity with α_1_. The β-subunit isoforms are more diverse. Compared to β_1_, β_2_ shows 39% identity and β_3_ is 36%. Seven different FXYD proteins have been identified (FXYD1 to FXYD7), which show 43 ~ 51% identity in their amino acid sequence.

The α- and β-subunits are expressed in a tissue specific manner (Herrera et al., 1987; Orlowski and Lingrel, 1988; Sweadner, 1989; Blanco et al., 1998; Benarroch, 2011). The α_1_-subunit is present in most tissues and the highest expression is found in kidney, brain and heart. Compared to the broad tissue distribution of the α_1_-subunit, the expression of other α-subunit isoforms is more restricted. The α_2_-subunit predominates in skeletal muscle, brain and heart; the α_3_-subunit is abundant in nervous tissue and heart. The α_4_-subunit was discovered as a testis-specific subunit (Shamraj and Lingrel, 1994), but now has also been found in adult human and mouse skeletal muscle (Keryanov and Gardner, 2002). Within the nervous system, the α-subunit isoforms are expressed in a cell type specific manner. For example, the α_1_-subunit is expressed in both neurons and glial cells, the α_2_-subunit is primarily found in astrocytes and oligodendrocytes and the α_3_-subunit is abundant in neurons (Benarroch, 2011). Like the α_1_-subunit, the β_1_-subunit is ubiquitously expressed in almost all tissues with the greatest levels in brain and kidney (Orlowski and Lingrel, 1988). The β_2_-subunit is mainly found in nervous tissue, kidney and heart (Avila et al., 1998), whereas the β_3_-subunit predominates in kidney and adrenal gland but is also presents in brain, lung and liver (Malik et al., 1998). The FXYD proteins show a tissue-specific distribution as well. FXYD1 (phospholemman) is expressed in heart and skeletal muscle; FXYD2 (γ-subunit) in kidney; FXYD3 (MAT-8) in stomach and colon; FXYD4 (CHIF) in kidney and colon; FXYD5 (dysadherin) in intestine, lung and kidney; FXYD6 and FXYD7 in brain (Garty and Karlish, 2006; Geering, 2006). In addition, the expression levels of α- and β-subunit change during development. For example, in brain, all three α-subunit isoforms increase in abundance from fetus to adult (Orlowski and Lingrel, 1988). However, the α_1_-subunit is more abundant in fetal kidney and heart than in adult tissues (Herrera et al., 1987). In rat heart, the α_3_-subunit decreases sharply after birth and is replaced by α_2_-subunit in adults (Zahler et al., 1996). The β_1_-subunit increases in the early stage of embryonic development, but decreases to the basal level during embryo implantation (Deng et al., 2013). With these diverse tissue distributions of Na,K-ATPase subunit isoforms, multiple Na,K-ATPase isozymes can form, rendering cells able to respond more precisely to extracellular stimuli. For instance, while in kidney α_1_β_1_ is the major αβ complex, all combinations of αβ complexes have been found in heart and brain (Mobasheri et al., 2000).

The spatial and temporal control of Na,K-ATPase expression occurs at the transcriptional, post-transcriptional, translational and post-translational level. Here, we will review findings on the transcriptional regulation of Na,K-ATPase over the past decades (Table 1). Extracellular stimuli, such as growth factors play a key role in the regulation of Na,K-ATPase by activating intracellular signaling pathways including protein kinase A (PKA), protein kinase C (PKC), and other Ca^2+^-regulated signaling pathways. Numerous DNA-binding-site-specific transcription factors have been reported to be involved in the regulation of Na,K-ATPase expression, including hormone receptors, Snail1, specificity protein (Sp) and Zinc finger E-box-binding homeobox 1 (ZEB1). Recently, it has been shown that hypermethylation in promoter regions of human *ATP1B1* gene is associated with reduced expression of β_1_-subunit (Selvakumar et al., 2014), indicating that epigenetic modification might be another mechanism of transcriptional regulation of Na,K-ATPase subunits.

**Table 1:** **Examples of factors that transcriptionally regulate Na,K-ATPase**.

**Regulator**	**The roles in regulating Na,K-ATPase transcription**	**References**
11-Dehydrocorticosterone	Increased α_1_ and β_1_ mRNA in vascular smooth muscle cells	Muto et al., 1998a
8-Bromo-cAMP	Increased α_1_ and β_1_ mRNA in a rat kidney epithelial cell line	Whorwood and Stewart, 1995
Aldosterone	Increased α_1_ and β_1_ mRNA in A6 kidney cells from Xenopus laevis	Verrey et al., 1987, 1988, 1989
	Increased α_1_ and β_1_ mRNA in adult and neonatal rat cardiocytes	Ikeda et al., 1991
	Increased α_1_ and β_1_ mRNA in vascular smooth muscle cells	Oguchi et al., 1993
	Increased α_3_ mRNA in rat hippocampus	Farman et al., 1994
	Increased α_1_ and β_1_ mRNA in rat vascular smooth muscle cells	Muto et al., 1996
	Increased α_1_ in rat cortical collecting duct	Tsuchiya et al., 1996
	Increased α_3_ and β_1_ in hippocampus, gyrus dentatus and periventricular gray substance	Grillo et al., 1997
	Increased α_1_ mRNA in the renal cortex	Seok et al., 1999
	Increased β_1_ mRNA in alveolar type 2 (AT2) cells	Olivera et al., 2000
	Increased α_1_, but not β_1_ mRNA in cortical collecting duct cells	Blot-Chabaud et al., 2001
	Increased α_2_and β_1_ mRNA in human skeletal muscle	Phakdeekitcharoen et al., 2011
Ammonia	Increased α_2_, but not α_1_ mRNA, decreased α_3_ mRNA	Xue et al., 2010
Angiotensin II	Increased α_1_ and β_1_ mRNA	Isenovic et al., 2004
Betamethasone	Increased α_1_ and β mRNA level in 10-day-old rats, but not in adult rats	Celsi et al., 1991
	Increased α_1_, α_2_, β_1_, but not α_3_ mRNA in infant rat heart	Wang and Celsi, 1993
	Increased α_1_ and β_1_ mRNA in infant rat kidney	Wang et al., 1994
C peptide	Increased α_1_ mRNA in human renal tubular cells	Galuska et al., 2011
Caffeine	Decreased α_1_ and β_1_ mRNA in rat kidney	Lee et al., 2002
Cholera toxin	Increased β_1_, but not α_1_ mRNA in a rat kidney epithelial cell line	Whorwood and Stewart, 1995
Corticosterone	Increased α_1_ and β_1_ mRNA in vascular smooth muscle cells	Muto et al., 1998a
Cyclic stretch	Increased α_1_and α_2_ mRNA in aortic smooth muscle cells	Sevieux et al., 2001
db-cAMP	Increased α_1_, but not β_1_ mRNA	Dagenais et al., 2001
Dexamethasone	Increased α_2_, but not α_1_, α_3_, and β mRNA in cultured neonatal rat cardiac myocytes	Orlowski and Lingrel, 1990
	Increased α_1_ and β_1_ mRNA in a rat liver cell line	Bhutada et al., 1991
	Increased α_1_ and β_1_ mRNA in fetal rat lung epithelial cell line	Chalaka et al., 1999
	Increased α_3_ and β_1_ mRNA in rat spinal cords	González et al., 1994, 1996
	Increased α_1_ and β_1_ mRNA in rat vascular smooth muscle cells	Muto et al., 1996
	Increase β_1_, but not α_1_ mRNA in alveolar epithelial type II cells	Barquin et al., 1997
	Increased β_1_, but not α_1_ mRNA in fetal lungs	Ingbar et al., 1997
	Increased α_1_ and β_1_ mRNA in a fetal rat lung epithelial cell line	Chalaka et al., 1999
	Increased β_1_, but not α_1_ mRNA in rat alveolar epithelial cells	Dagenais et al., 2001; Hao et al., 2003a,b
	Decreased α_1_ mRNA in rat capsule-epithelium of lenses	Xie and Askari, 2002
DNA methylation	Decreased α_3_, β_1_ and *FXYD1* mRNA	Henriksen et al., 2013; Selvakumar et al., 2014
Dopamine	Increased β_1_ mRNA in rat alveolar epithelial cells	Guerrero et al., 2001
EGF	Increased α_1_and β_1_ mRNA in alveolar epithelial cells	Danto et al., 1998
	Increased α_2_ but not α_1_ mRNA in primary cultures of mouse astrocytes	Xue et al., 2010
Elevated Ca^2+^	Increased α_1_ and β_1_ mRNA in rat kidney	Rayson, 1991
Elevated intracellular Na^+^	Increased α_1_ and β_1_ mRNA in rat kidney epithelial cells	Muto et al., 2000
FGF	Increased α_1_ and β_1_ mRNA in vascular smooth muscle cells	Nemoto et al., 1997
Forskolin	Increased α_1_ and β_1_mRNA in a rat kidney epithelial cell line	Whorwood and Stewart, 1995
Glucose	Increases α_1_ and β_1_ mRNA	Muto et al., 1998a
Glycyrrhetinic acid	Decreased α_1_ and β_1_ mRNA in rat kidney epithelial cells	Whorwood and Stewart, 1995
High-fat diet	Increased α_1_ mRNA in nuclear extracts from gastrocnemius muscle	Galuska et al., 2009
Hyperoxia	Selectively increased β_1_ mRNA in MDCK cells	Wendt et al., 1998
Hypoxia	Down-regulated the expression of Na,K-ATPase in alveolar cells, renal proximal tubule cells and lung cancers	Planes et al., 1997; Adachi et al., 2004; Yu and Hales, 2011
IL-2	Increased α_1_ and β_1_ mRNA in human blood lymphocytes	Karitskaya et al., 2010
Insulin	Increased α2, not α1 mRNA, decreased β1 mRNA in 3T3-L1 cells	Russo and Sweadner, 1993
	Increased α2, not α1 mRNA in VSMC cells	Tirupattur et al., 1993
Ischemia and reflow	Decreased α_1_ and β mRNA level in rat kidney	Van Why et al., 1994
KCl	Increased α_1_, α_3_, and β_1_ mRNA in neurons	Johar et al., 2012, 2014
KGF	Increased α_1_, but not β_1_ mRNA in alveolar type II cells	Borok et al., 1998
Low K^+^	Increased α_1_ and β_1_ mRNA in cultured renal proximal tubule cells	Tang and McDonough, 1992
	Increased α_1_ and β_1_ mRNA in rat cardiac myocytes	Qin et al., 1994; Zhuang et al., 2000; Wang et al., 2007b
Mannitol	Increased α_1_and β_1_ mRNA	Muto et al., 1998b
Manganese	Decreased α_3_ mRNA in mice	Wang et al., 2013
Nitric oxide	Decreased α_1_ mRNA in medullary thick ascending limb of Henle (MTAL) cell lines	Kone and Higham, 1999
NRF1	Increased β_1_ but decreased α_1_ mRNA	Johar et al., 2012
Ouabain	Increased α_1_ and β_1_ mRNAs in cultured rat astrocytes	Hosoi et al., 1997
	Increased α_1_ and β_1_ mRNA in rat kidney epithelial cells	Muto et al., 2000
	Regulated α_3_ and β_1_ mRNA in cultured neonatal rat cardiac myocytes	Kometiani et al., 2000
Pertussis toxin	Increased α_1_ and β_1_ mRNA in a rat kidney epithelial cell line	Whorwood and Stewart, 1995
PHA	Increased α_1_ and β_1_ mRNA in human blood lymphocytes	Karitskaya et al., 2010
Progesterone	Increased β_1_ mRNA	Cochrane et al., 2012
	Increased β_1_ mRNA in mouse uterus	Deng et al., 2013
Prostaglandin E1	Increased α and β mRNA in MDCK cells	Taub et al., 1992, 2004; Matlhagela and Taub, 2006
	Increased α and β mRNA in rabbit renal proximal tubule cells	Herman et al., 2010
Prostaglandin E2	Increased *ATP1B1* promoter activity in rabbit renal proximal tubule cells	Herman et al., 2010
Serum	Increased α_1_ and β_1_ mRNA in a rat liver cell line, Clone 9	Kirtane et al., 1994
	Increased α_1_ and β_1_ mRNA in vascular smooth muscle cells	Nemoto et al., 1997
Snail1	Selectively repressed β_1_, but not α_1_ mRNA in MCF7 and MDCK cells	Espineda et al., 2004
Sp (Sp1, Sp3, Sp4)	Increased α_1_, α_3_, and β_1_ mRNA in murine neurons	Johar et al., 2014
T3	Increased α and β mRNA in rat kidney cortex	Gick and Ismail-Beigi, 1990
	Increased α, but not β mRNA in rat liver	Gick and Ismail-Beigi, 1990
	Increased α and β mRNA in rat kidney	McDonough et al., 1988
	Increased α_2_, α_3_, and β, but not α_1_ mRNAs in neonatal rat cardiac myocytes	Orlowski and Lingrel, 1990
	Increased α_1_ and β mRNA in a rat liver cell line Clone 9	Gick and Ismail-Beigi, 1990
	Increased α_1_, α_3_, and β_1_ mRNA in neonatal rat myocardium	Melikian and Ismail-Beigi, 1991
	Increased α_1_, α_2_, and β_1_ mRNA in cardiac myocytes	Hensley et al., 1992
	Increased α_1_, α_2_, α_3_, and β_1_ mRNA in cultured neonatal rat cardiocytes	Kamitani et al., 1992
	Increased α_2_ and β_2_ mRNA in skeletal muscle	Azuma et al., 1993
	Increased α_1_ and β_1_ mRNA in cultured rat mesangial cells	Ohara et al., 1993
	Increased α_1_ and β_1_ mRNA in in rat jejunum and Caco-2 cells	Giannella et al., 1993
	Increased α_2_ mRNA in neonatal rat cardiac myocytes	Huang et al., 1994
	Increased α and β mRNA in rabbit renal proximal tubule cells	Lin and Tang, 1997
	Increased α_1_, α_2_, and α_3_ mRNA in rat brain	Bajpai and Chaudhury, 1999
	Increased α_2_ and β_1_ mRNA in rat heart	Shao et al., 2000
	Increased α_2_ and β_1_ mRNA in human skeletal muscles	Phakdeekitcharoen et al., 2007
T4	Increased α_1_, α_2_, and β_1_ mRNA in rat heart	Shao et al., 2000
Tetrodotoxin	Decreased α_1_, α_3_, *and* β*_1_* mRNA in murine neurons	Johar et al., 2012, 2014
TGF-β_1_	Decreased β_1_, α_1_, α_2_, and α_3_ mRNA in young FRTL-5 cells	Pekary et al., 1997
TGF-β_2_	Selectively decreased β_1_ mRNA in ARPE-19 cell	Mony et al., 2013
Uremia	Decreased α_1_, but increased α_2_ in rat skeletal muscle	Bonilla et al., 1991
Vasopressin	Increased α_1_, but not β_1_ mRNA in cortical collecting duct cells	Blot-Chabaud et al., 2001
Veratridine (a Na^+^ channel activator)	Increased α_1_ and β_1_ mRNA in rat vascular smooth muscle cells	Yamamoto et al., 1994

## Promoter analysis of Na,K-ATPase α- and β-subunits

**Figure 1:**
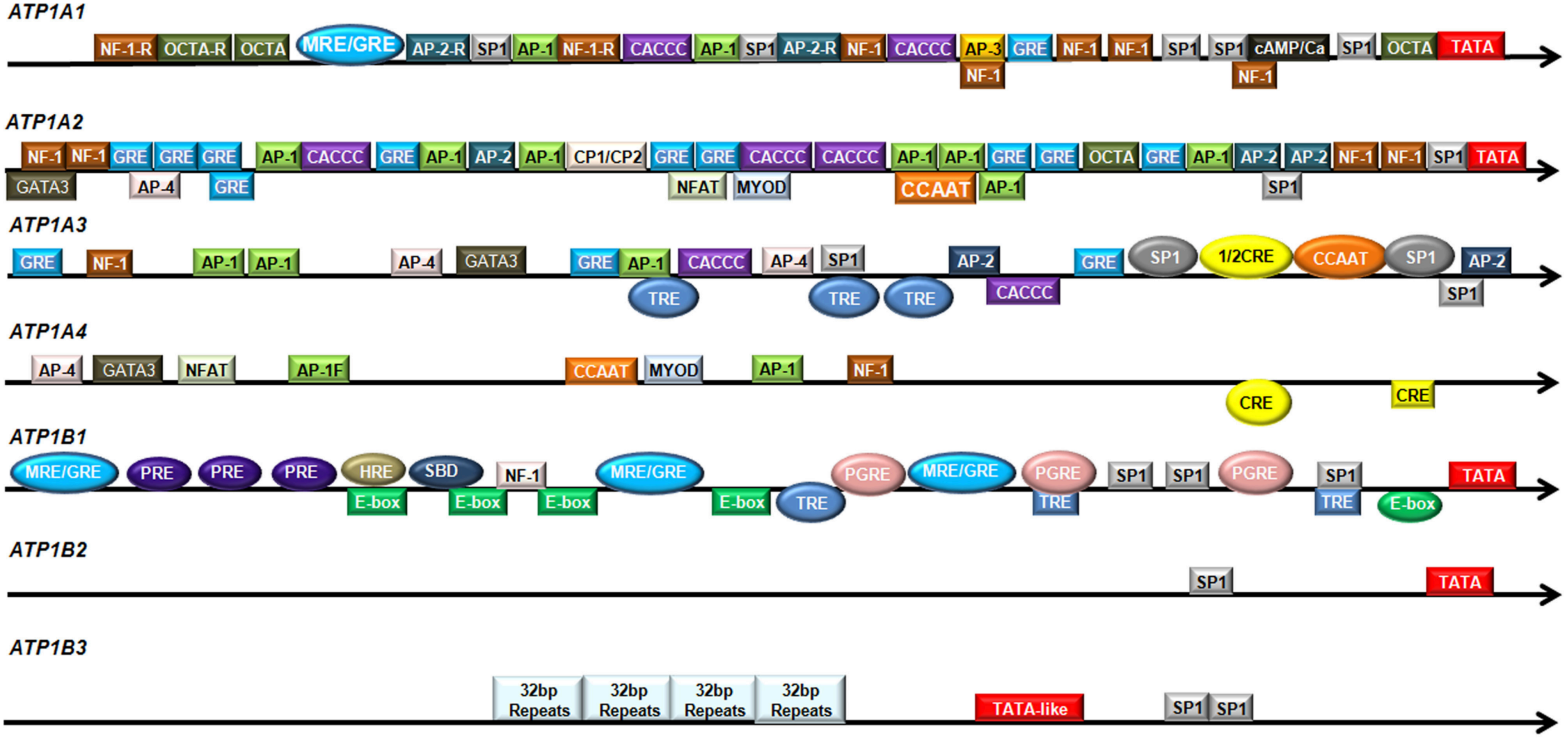
**Regulatory elements in the promoter regions of human Na,K-ATPase subunits**. The promoter regions of human Na,K-ATPase are shown in the direction from 5′ to 3′. The colored boxes indicate potential transcription factor binding sites. The colored ovals represent experimentally verified transcription factor binding sites.

Transcription factors activate or repress Na,K-ATPase expression by binding to specific DNA elements on the promoter regions. There are numerous sequence-specific elements on the promoter regions of the various Na,K-ATPase subunits, of which β_1_ has been extensively studied (Figure 1).

### α_1_-subunit

The α_1_-subunit is encoded by the gene *ATP1A1*. The *ATP1A1* promoter has very high GC content, which is a common characteristic of all housekeeping genes. It contains a potential TATA box (−27 bp; Shull et al., 1990), two mineralocorticoid/glucocorticoid response element (MRE/GRE) sites (−598 to −574 bp and −252 bp; Kolla et al., 1999); potential binding sites for AP-1(−507 and −453 bp), AP-2 (−568 and −380 bp), AP-3 (−286 bp), and several CTF/NF-1 and potential Sp1 binding sites (Shull et al., 1990). The nucleotide sequence from around −100 bp to the transcription initiation site is highly conserved among the human and rat *Atp1a1* genes (Kobayashi et al., 1997). An asymmetrical ATF/CRE site (−77 to −64 bp) is found in the rat *Atp1a1* promoter (Kobayashi and Kawakami, 1995). In addition, the rat *Atp1a1* promoter contains a Sp1 consensus sequence (−56 to −51 bp) and a Sp1 binding site (−120 to −106 bp; Suzuki-Yagawa et al., 1992).

### α_2_-subunit

The α_2_-subunit is encoded by the gene *ATP1A2*. The promoter region of human *ATP1A2* contains a potential TATA box, two potential Sp1 recognition sites, numerous potential AP-1, AP-2, and NF-1 binding sites, and several sequences that are similar to the glucocorticoid receptor binding site (Shull et al., 1989; Malyshev et al., 1991). The major positive regulatory elements are located at the position from −175 to −108 bp on the rat *Atp1a2* gene, harboring two E boxes (−144 to −139 bp and −135 to −130 bp), a Spl binding consensus sequence (−123 to −118 bp) and a Sp1 binding site (−114 to −109 bp; Ikeda et al., 1993).

### α_3_-subunit

The α_3_-subunit is encoded by the gene *ATP1A3*. The human *ATP1A3* promoter has high GC content, but no conventional TATA box (Malyshev et al., 1991). Sequence analysis reveals several potential Sp-1 binding sites and AP-1 sites, two AP-2 sites, two AP-4 sites, three GRE elements, two CACCC sequences, a NF-1 binding site, a couple of TRE elements (Pathak et al., 1990), and the binding sites for CREB/ATF and NF-Y/C-EBP (Benfante et al., 2005). These sites are well conserved between the human and rat *Atp1a3* gene (Pathak et al., 1990). It was subsequently shown that two Sp1 binding sites (−110 to −100 bp; −59 to −47 bp), a CCAAT box (−64 to −61 bp) and a half CRE-like site (−87 to −83 bp) are functional. Mutations in the Sp1 binding site (−110 to −100 bp) or the CCAAT box significantly reduced the *ATP1A3* promoter activity and these two sites worked synergistically in inducing *ATP1A3* promoter activity. The CRE-like element itself did not affect *ATP1A3* promoter activity, but cooperated with the upstream Sp1 site (Benfante et al., 2005). Three TRE elements (−636 to −457 bp; −218 to −106 bp; and −106 to −6 bp) showed strong and specific interaction with thyroid hormone receptor (TR) and modulated *ATP1A3* promoter activity (Bajpai et al., 2001). In addition, the region from −210 bp to the transcription initiation site was sufficient to direct brain-specific expression of the rat α_3_-subunit (Pathak et al., 1994).

### α_4_-subunit

The α_4_-subunit is encoded by the gene *ATP1A4*. In silicon sequence analysis of putative promoter regions of human and mouse *ATP1A4* showed that they do not contain a TATA-box sequence, but have potential binding sites for transcription factors AP-1, AP-4, GATA3, NF-Y, MYOD, NF-1, NFAT (Keryanov and Gardner, 2002) and two consensus CRE sites (Rodova et al., 2006). However, *ATP1A4* does not have any Sp1 binding site that is common on the promoter regions of other subunits (Keryanov and Gardner, 2002).

### β_1_-subunit

The β_1_-subunit is encoded by the gene *ATP1B1*. It contains three potential MRE/GRE half sites (−1048 to −1027 bp; −650 to −630 bp; −276 to −271 bp; Derfoul et al., 1998), four E-boxes (−752 to −747 bp; −708 to −703 bp; −654 to −649 bp; −509 to −504 bp), a non-canonical E-box (−71 to −66 bp), a NF-1 binding site (−657 to −653 bp; Espineda et al., 2004), three prostaglandin response elements (PGRE: −445 to −438 bp; −226 to −216 bp; −100 to −92 bp; Matlhagela et al., 2005; Matlhagela and Taub, 2006), a putative hypoxia response element (HRE: −750 to −746 bp; Mony et al., 2013), a Smad binding domain (SBD: −728 and −721 bp; Mony et al., 2013), three thyroid hormone response elements (TRE; Feng et al., 1993), three progesterone element (PRE) half sites (Cochrane et al., 2012) and several Sp1 binding sites (Matlhagela et al., 2005; Matlhagela and Taub, 2006). Furthermore, the mouse *Atp1b1* promoter region also contains a Cebpb binding site (Deng et al., 2013). The 5′ flanking region of rat *Atp1b1* shows 68% homology with human *ATP1B1*. It contains a consensus TATA box sequence (−31 bp), two CAAT boxes, four GC boxes, five half-site TREs, several GRE and calcium and serum response elements (Liu and Gick, 1992).

### β_2_-subunit

The β_2_-subunit is encoded by the gene *ATP1B2*. The region from −280 bp to the transcriptional initiation site on the promoter of *ATP1B2* gene is highly conserved between rat, mouse and human. The human *ATP1B2* promoter contains a consensus TATA sequence at position −94 bp and a Sp1 binding site at position −120 bp (Avila et al., 1998). The mouse *Atp1b2* promoter has a TATA-like sequence (−23 to −28 bp), a putative CAAT box (−137 to −142 bp), and a GC box (−144 to −149 bp; Shyjan et al., 1991). Sequence analysis revealed a potential TATA box (−29 bp); two Sp1 binding sites (−55 and −147 bp) and a serum response element (SRE: −263 bp) on the rat *Atp1b2* promoter region (Kawakami et al., 1990). In addition, an AMOG regulatory element (AMRE: GAGGCGGGG at position −87 to −79 bp) and a functional Sp1 binding site (−147 to −142 bp) were found in rat *Atp1b2* promoter regions (Kawakami et al., 1992, 1993).

### β_3_-subunit

The β_3_-subunit is encoded by the gene *ATP1B3*. The human *ATP1B3* promoter contains a TATA-like sequence (−413 to −408 bp) and two Sp1-like elements (−323 to −315 bp and −312 to −304 bp). Moreover, the human *ATP1B3* also has four identical 32-nucleotide-long repeats from position −760 to −633 bp. The sequence from −1 to −328 bp on the 5′ flanking region of human and mouse *Atp1b3* shows high homology. The mouse *Atp1b3* promoter has one Sp1-like sequence (−319 to −310 bp), but does not have any TATA-like sequence. No CAAT or GC boxes are present at the promoter region of human or mouse *Atp1b3* (Malik et al., 1998).

## Transcriptional regulation of Na,K-ATPase by hormones and nuclear receptors

Hormones are major regulators of Na,K-ATPase expression. Mineralocorticoids (Verrey et al., 1987, 1988, 1989; Ikeda et al., 1991; Oguchi et al., 1993; Farman et al., 1994; Muto et al., 1996; Tsuchiya et al., 1996; Grillo et al., 1997; Seok et al., 1999; Olivera et al., 2000; Blot-Chabaud et al., 2001; Phakdeekitcharoen et al., 2011), glucocorticoids (Orlowski and Lingrel, 1990; Bhutada et al., 1991; Celsi et al., 1991; González et al., 1994, 1996; Muto et al., 1996; Barquin et al., 1997; Ingbar et al., 1997; Chalaka et al., 1999; Dagenais et al., 2001; Hao et al., 2003a,b), thyroid hormone (McDonough et al., 1988; Gick and Ismail-Beigi, 1990; Orlowski and Lingrel, 1990; Melikian and Ismail-Beigi, 1991; Hensley et al., 1992; Kamitani et al., 1992; Azuma et al., 1993; Giannella et al., 1993; Ohara et al., 1993; Huang et al., 1994; Lin and Tang, 1997; Bajpai and Chaudhury, 1999; Shao et al., 2000; Phakdeekitcharoen et al., 2007), insulin (Russo and Sweadner, 1993; Tirupattur et al., 1993; Sweeney and Klip, 1998), progesterone (Cochrane et al., 2012; Deng et al., 2013), androgen (Blok et al., 1999), and vitamin D3 (Billecocq et al., 1997) have been reported to activate or repress Na,K-ATPase transcription. These hormones bind to and activate their receptors, which belong to the family of nuclear receptors with C4 zinc-fingers that then translocate into the nucleus to bind to the promoter regions of the target genes to regulate gene expression.

### Mineralocorticoid receptor (MR) and glucocorticoid receptor (GR)

MR and GR are members of the nuclear receptor family. They have three major functional domains: a N-terminal regulatory domain (NTD); a DNA-binding domain (DBD) and a ligand-binding domain (LBD; Kumar and Thompson, 2005). The MR is highly homologous to the GR in the DBD and LBD but not in the NTD (Kolla et al., 1999) and while mineralocorticoids only activate the MR, glucocorticoids activate both GR and MR. The activated MR and GR translocate into the nucleus and bind to a specific palindromic DNA sequence known as glucocorticoid/mineralocorticoid response element (GRE/MRE) located in the promoter regions of target genes, thus modulating gene transcription. The GRE is composed of imperfect inverted hexanucleotide repeats separated by three nucleotides (GGTACA NNN TGTTCT; Nordeen et al., 1990).

Glucocorticoids regulate the transcription of Na,K-ATPase in a variety of tissues including lung, kidney, liver, heart, spinal cord, cardiac muscle and smooth muscle (Orlowski and Lingrel, 1990; Bhutada et al., 1991; Celsi et al., 1991; Wang and Celsi, 1993; González et al., 1994, 1996; Wang et al., 1994; Muto et al., 1996; Barquin et al., 1997; Ingbar et al., 1997; Chalaka et al., 1999; Dagenais et al., 2001; Hao et al., 2003b). This regulation is specific to the cell type and the developmental stage. For example, dexamethasone, a synthetic potent glucocorticoid, increased the mRNA levels of α_1_- and β_1_-subunit in a rat liver cell line and in a fetal rat lung epithelial cell line (Bhutada et al., 1991; Chalaka et al., 1999). However, in cultured neonatal rat cardiac myocytes, dexamethasone only induced α_2_-subunit mRNA, and α_1_-, α_3_-, and β-subunit transcripts remained unchanged (Orlowski and Lingrel, 1990). Injection of betamethasone, another synthetic analog of glucocorticoid, increased α_1_- and β-subunit mRNA abundance in 10-day-old rats, but not in adult rats (Celsi et al., 1991).

Mineralocorticoids, like aldosterone, also regulate the mRNA levels of Na,K-ATPase in diverse tissues such as kidney, heart, brain, vascular smooth muscle and skeletal muscle (Verrey et al., 1987, 1988, 1989; Ikeda et al., 1991; Oguchi et al., 1993; Farman et al., 1994; Tsuchiya et al., 1996; Grillo et al., 1997; Seok et al., 1999; Olivera et al., 2000; Blot-Chabaud et al., 2001; Phakdeekitcharoen et al., 2011). Aldosterone increased both α_1_- and β_1_-subunit transcripts in kidney, heart and vascular muscles and this up-regulation of β_1_-subunit was much stronger than that of α_1_-subunit (Verrey et al., 1987, 1988, 1989; Ikeda et al., 1991; Oguchi et al., 1993). Aldosterone also increased α_2_- and β_1_-subunit mRNA in human skeletal muscle (Phakdeekitcharoen et al., 2011). In addition, aldosterone selectively up-regulated α_3_- and β_1_-subunit mRNA in some brain regions, such as hippocampus, gyrus dentatus and periventricular gray substance (Farman et al., 1994; Grillo et al., 1997). The transcriptional regulation of α_1_- and β_1_-subunit by aldosterone was mediated mostly by MR (Muto et al., 1996; Kolla et al., 1999), whereas dexamethasone-mediated induction of β_1_-subunit mRNA occurred through GR (Muto et al., 1996). Inhibiting GR and MR activities by their specific antagonists also reduced α_1_- and β_1_-subunit levels (Whorwood et al., 1994; Hao et al., 2003b). In monkey kidney CV-1 cells, which have no detectable MR and GR, activation of exogenous MR or GR alone by aldosterone or triamcinolone acetonide increased β_1_-subunit gene promoter activity. However, activation of both receptors inhibited transcription from the β_1_-subunit gene promoter (Derfoul et al., 1998) and the N-terminal region of MR mediated this inhibitory effect (Derfoul et al., 2000).

The promoter region of the *ATP1B1* gene contains three potential MRE/GREs at nucleotide position −1048 to −1027 bp; −650 to −630 bp and −276 to −271 bp. The first two contain two half-binding sites separated by 10 and 9 base pairs, respectively, whereas the last one consists of a single half-site. Although all three MRE/GREs were involved in the induction of *ATP1B1* promoter activity by corticoids, the one at position −650 to −630 bp was the strongest (Derfoul et al., 1998). Both MR and GR bound to the GRE/MRE (−650 to −630 bp) and activated the transcription from a heterologous neutral promoter in response to corticoids (Derfoul et al., 1998; Hao et al., 2003b). A similar element was found in the human *ATP1A1* promoter region at position −598 to −574 bp in which two half sites are separated by 12 base pairs. This MRE/GRE bound both MR and GR and activated a heterologous neutral promoter activity (Kolla et al., 1999). In addition, a GRE (+434 to +448 bp) in the coding region of the rat *Atp1b1* gene has been reported in a rat lung epithelial cell line. This element acted as an enhancer and increased the transcription from a heterologous promoter by dexamethasone (Hao et al., 2003a).

### Progesterone receptor

Progesterone receptor (PR) also belongs to the nuclear receptor family. PR's sequence and architecture is very similar to MR and GR (Leo et al., 2004). Like MR and GR, PR is activated by its ligand progesterone and then translocates into the nucleus, forms a homodimer and then binds to specific progesterone response elements (PREs) within the promoter of target genes to modulate transcription (Leonhardt et al., 2003). Progesterone increased the mRNA and protein levels of β_1_-subunit (Cochrane et al., 2012; Deng et al., 2013), and this up-regulation was enhanced by estradiol-17β (Deng et al., 2013). RU486, a PR antagonist, completely blocked the increase in β_1_-subunit mRNA by progesterone (Deng et al., 2013). In addition, human β_1_-subunit was up-regulated by both PR-A and PR-B, two different PR isoforms (Richer et al., 2002). Moreover, PR bound to three PRE half sites on the *ATP1B1* promoter; Progestin, a synthetic progestogen with similar effect to progesterone, induced *ATP1B1* promoter activity, an induction that was suppressed by RU486 (Cochrane et al., 2012).

### Thyroid hormone receptor

Thyroid hormone receptor (TR) is a member of the nuclear receptor superfamily and is activated by thyroid hormone, triiodothyronine (T3) and its prohormone, thyroxine (T4). Although TR alone can bind as monomer or homodimer to specific DNA sequences in the gene promoter regions known as thyroid hormone response elements (TREs), it preferentially forms heterodimers with retinoid X receptor (RXR) and then binds to TREs, thus enhancing or inhibiting the expression of genes that are involved in development, homeostasis, cellular proliferation and differentiation (Wu and Koenig, 2000). T3 has been shown to regulate the abundance of Na,K-ATPase mRNA and protein in heart, kidney, liver, intestines, brain, and skeletal muscle among others (McDonough et al., 1988; Gick and Ismail-Beigi, 1990; Orlowski and Lingrel, 1990; Melikian and Ismail-Beigi, 1991; Hensley et al., 1992; Kamitani et al., 1992; Azuma et al., 1993; Giannella et al., 1993; Ohara et al., 1993; Huang et al., 1994; Lin and Tang, 1997; Bajpai and Chaudhury, 1999; Shao et al., 2000; Phakdeekitcharoen et al., 2007). In general, T3 up-regulated the transcription of Na,K-ATPase subunits. For example, T3 increased the mRNA levels of α_1_-, α_2_-, α_3_-, and β_1_-subunit in cultured neonatal rat cardiocytes (Kamitani et al., 1992) and α_2_-, β_1_-, and β_2_-subunit in skeletal muscle (Azuma et al., 1993; Phakdeekitcharoen et al., 2007). In hypothyroid rat brain, the mRNA level of α_1_-, α_2_-, and α_3_-subunit was reduced during the first 3 weeks of brain development (Chaudhury et al., 1996). The transcription of these mRNAs was decreased in nuclei from hypothyroid cerebra and pretreatment with T3 increased the rates of transcription (Bajpai and Chaudhury, 1999). The promoter regions of *Atp1a1, Atp1a2, Atp1a3*, and *ATP1B1* genes have TRE sequences (Kamitani et al., 1992; Feng et al., 1993; Giannella et al., 1993). In addition, two negative thyroid receptor elements (−116 and −6 bp and −6 and +80 bp) were found in rat *Atp1a3* promoter. The TR/RXR complex bound to these elements and inhibited T3-mediated gene expression in neonatal rat cardiac myocytes, which only occured with prolonged incubation with T3 (He et al., 1996).

### Insulin

Insulin is an important regulator of Na,K-ATPase. It binds to insulin receptor and activates various cellular signaling pathways, thus regulating cell functions. Insulin modulates Na,K-ATPase activity by increasing Na^+^ influx; increasing the affinity of Na,K-ATPase for intracellular Na^+^; altering phosphorylation status of Na,K-ATPase and facilitating the translocation of intracellular Na,K-ATPase to the plasma membrane. In addition, insulin also regulates the transcription, translation and stability of Na,K-ATPase (Ewart and Klip, 1995; Lavoie et al., 1996; Sweeney and Klip, 1998; Therien and Blostein, 2000; Clausen, 2003). The transcriptional regulation of Na,K-ATPase by insulin occurs in an isoform specific manner. For example, insulin selectively increased α_2_, but not α_1_ mRNA level in vascular smooth muscle cells (VSMC) and 3T3-L1 cells (Russo and Sweadner, 1993; Tirupattur et al., 1993; Sweeney and Klip, 1998). However, it reduced the β_1_ mRNA level (Russo and Sweadner, 1993).

## Transcription factors regulating Na,K-ATPase

### Specificity protein

Specificity protein (Sp) is a small family of zinc-finger transcription factors with four members: Sp1, Sp2, Sp3, and Sp4. Sp1, Sp2, and Sp3 are ubiquitously expressed, while Sp4 is expressed mainly in neurons and testes. The Sp family members contain three zinc fingers close to the C-terminus, which is DBD, and preferentially bind to the GC box [(G/T)(G/A)GGC(G/T)(G/A)(G/A)(G/T)] on the promoter regions of target genes to enhance gene expression. Sp1, Sp3, and Sp4 have a highly conserved DBDs and they recognize the same consensus GC rich element with identical affinities. However, Sp2, in which the important histidine residue within the first zinc finger is replaced by a leucine residue, does not bind to the GC box but instead binds to a GT rich element (Suske, 1999). Potential Sp1 binding sites were found on the promoter regions of almost all Na,K-ATPase subunits, including *Atp1a1, Atp1a2, Atp1a3, Atp1b1*, and *Atp1b2* (Shull et al., 1989, 1990; Kawakami et al., 1990; Yagawa et al., 1990; Malyshev et al., 1991; Suzuki-Yagawa et al., 1992; Ikeda et al., 1993; Yu et al., 1996; Avila et al., 1998; Benfante et al., 2005). It has been shown that Sp1 and Sp3 bind to GC boxes on the promoter regions of rat *Atp1a1* (Kobayashi et al., 1997) and *Atp1b1* (Murakami et al., 1997). Mutations in the GC box sequences decreased basal *Atp1b1* transcription in neonatal rat cardiac myocytes (Zhuang et al., 2000). Sp1, Sp3 and Sp4 also bind to two Sp1 binding sites (−110 to −100 bp; −59 to −47 bp) on human *ATP1A3* promoter. Mutations in Sp1 binding site (−110 to −100 bp) significantly reduced the promoter activity (Benfante et al., 2005). Recently, Sp1, Sp3, and Sp4 had been shown to increase α_1_-, α_3_-, and β_1_-subunit transcription in murine neurons by binding to their promoter regions (Johar et al., 2012). Mutating the Sp binding sites on *Atp1a1, Atp1a3, and Atp1b1* promoters led to a significant decrease in their promoter activity. Silencing Sp1, Sp3, or Sp4 significantly decreased the mRNA levels of α_1_-, α_3_-, and β_1_-subunit, while overexpressing Sp1, Sp3, and Sp4 increased their transcription (Johar et al., 2012). Sp1 also binds to two Sp binding sites located near its TATA box on rat *Atp1b2* promoter and enhanced the expression of β_2_-subunit. In addition, the functional Sp-binding site on *Atp1a1* promoter is well conserved in rat brain, kidney and liver (Suzuki-Yagawa et al., 1992; Kobayashi et al., 1997; Nemoto et al., 1997), indicating that it might contribute to the constitutional expression of α_1_-subunit. The Sp binding sites are also required for the regulation of Na,K-ATPase transcription in response to extracellular stimuli. For example, hyperoxia selectively up-regulated β_1_-subunit transcription in MDCK cells (Wendt et al., 1998). Hyperoxia increased the binding of Sp1 and Sp3 to the *Atp1b1* promoter and deletion of a GC rich element resulted in loss of both basal and hyperoxia-activated transcription (Wendt et al., 2000). Sp might also augment transcriptional activation of Na,K-ATPase through other transcription factors. For example, Sp1 significantly enhanced MR and GR expression (Kolla et al., 1999) and elevated MR and GR might synergize with Sp1 to upregulate Na,K-ATPase transcription.

### Snail

Snail1 is the first identified member of the Snail family, which belongs to the zinc-finger transcription factors and has three members in vertebrates: Snail1, Snail2, and Snail3. The Snail family has a highly conserved C-terminal region, which is the DBD containing four to six zinc fingers; and a divergent N-terminal region, which contains a SNAG (Snail/Gfi) domain and recruits the transcription repressor complex (such as sin3A/HDAC). The Snail family functions as a transcriptional repressor by binding to the E-box (CAGGTG) on the promoter regions of target genes which are important for embryonic development as well as tumor progression (Nieto, 2002). Four E-boxes and a non-canonical E-box are present on the promoter region of the *ATP1B1* gene. Snail1 binds to the non-canonical E-box located at position −71 to −66 bp and selectively repressed the expression of β_1_-subunit without affecting the α_1_-subunit level (Espineda et al., 2004). Overexpression of Snail1 repressed *ATP1B1* promoter activity in a dose dependent manner and markedly reduced the level of β_1_-subunit in MCF7 and MDCK cells. Knock-down of Snail by RNA interference increased the abundance of β_1_-subunit mRNA (Espineda et al., 2004).

### Activating transcription factor 1 (ATF-1)/cAMP response element binding protein (CREB) family

ATF/CREB belongs to the basic leucine zipper factor (bZIP) family, which bind to DNA as dimers and function as activators. The ATF/CREB family is composed of different ATFs, CREB, CREM (cAMP response element modulator) and related proteins. This family contains an N-terminal DBD and a C-terminal bZIP domain that binds other bZIP transcription factors to form homo- and heterodimers. Both ATF and CREB bind to the cAMP response element (CRE: GTGACGT A/C A/G). Phosphorylation of ATF-1 and CREB by multiple kinases enhances the binding to CRE and activates the transcription of target genes which play an important role in cell survival and cell growth (Hai and Hartman, 2001). Dibutyryl cAMP (db-cAMP), a cell-permeable cAMP analog that activates cAMP dependent protein kinase (PKA) and phosphorylates the CREB family, induced the mRNA of α_1_-subunit but not β_1_-subunit (Dagenais et al., 2001). The rat *Atp1a1* promoter has an asymmetrical ATF/CRE site at position −70 to −66 bp. The ATF-1/CREB heterodimer bound to this site and induced constitutive expression of α_*l*_-subunit. Blocking the binding of ATF-1/CREB to the CRE site reduced α_1_-subunit transcription (Kobayashi and Kawakami, 1995). Phosphorylated ATF-1 increased rat α_1_-subunit transcription and dephosphorylation of the ATF-1/CREB heterodimer by alkaline phosphatase reduced its binding to the CRE site on rat *Atp1a1* gene (Kobayashi et al., 1997). In addition, human *ATP1A4* promoter region has consensus CRE sites. db-cAMP and ectopic expression of CREMtau, a testis specific splice variant of CREM activated the *ATP1A4* promoter activity. This activation required the CRE site located at −263 bp relative to the transcription initiation site (Rodova et al., 2006).

### Zinc finger E-box binding homeobox 1 (ZEB1)

The ZEB family has two members in vertebrates: ZEB1 (also known as Areb6) and ZEB2. They contain five major domains: two zinc-finger clusters separated by a central homeodomain, a Smad interacting domain (SID) and a CtBP interacting domain (CID). Each zinc finger cluster contains 3 or 4 zinc fingers and is responsible for DNA binding. ZEB binds to E-boxes (CACCT and CACCTG) on the promoter of target genes and recruits co-activators (PCAF or p300), thus activating gene transcription (Vandewalle et al., 2009). The rat *Atp1a1* promoter has a consensus Areb6 (ZEB1) binding sequence and Areb6 (ZEB1) had been shown to activate gene transcription in a cell-specific manner (Watanabe et al., 1993). High-fat diet also induced the binding of ZEB to rat *Atp1*α*1* promoter and increased the mRNA and protein level of α_1_-subunit in nuclear extracts from gastrocnemius muscle (Galuska et al., 2009). Moreover, C-peptide increased the binding of ZEB to rat *Atp1*α*1* promoter region. Partial silencing of ZEB markedly reduced the effect of C-peptide on the increase in α_1_-subunit expression (Galuska et al., 2011). In addition, ZEB binding sites were also found on *FXYD1* promoter at positions −270 to −295 bp and −329 to −354 bp (Galuska et al., 2009).

### CCAAT box binding transcription factors (CBFs)

CCAAT box is one of the most common elements in eukaryotic promoters, which is often found between -60 to -100 bp relative to the transcription initiation site. Several transcription factors have been reported to bind to the CCAAT box, including NF-Y, NF-1, and CCAAT/enhancer-binding protein (C/EBP) isoforms (CEBP-α, CEBP-β, CEBP-γ, CEBP-δ, and CEBP-ε), which are essential for optimal transcriptional activation of target genes (Mantovani, 1998). CCAAT boxes have been found in *Atp1a2, Atp1a3*, and *Atp1b1* promoter regions (Pathak et al., 1990; Malyshev et al., 1991; Benfante et al., 2005; Deng et al., 2013). The CCAAT box (−61 to −64 bp) on human *ATP1A3* promoter is well conserved between rat and human and NF-YB had been shown to bind to this CCAAT box. The presence of the CCAAT box significantly increased *ATP1A3* promoter activity in both neuron and non-neuron cell lines, while mutations in the CCAAT box reduced the promoter activity (Benfante et al., 2005). In addition, the CCAAT/enhancer binding protein beta (Cebpb) might be involved in regulating β_1_-subunit expression in human luminal epithelial cells. The active isoform of Cebpb significantly increased *Atp1b1* promoter activity, but the truncated isoform of Cebpb had no effects on *Atp1b1* promoter activity (Deng et al., 2013).

## Growth factors and transcriptional regulation of Na,K-ATPase

### Transforming growth factor-β

Transforming growth factor-β (TGF-β) is a secreted protein that regulates numerous physiological processes, including cell differentiation, proliferation, development and survival. TGF-β has three isoforms in mammals: TGF-β_1_, TGF-β_2_, and TGF-β_3_, which are encoded by different genes. Aberrant TGF-β signaling has been linked to cancer initiation, invasion and metastasis (Lamouille et al., 2014). TGF-β treatment reduced mRNA and protein levels of Na,K-ATPase α- and β-subunits in primary cultures of renal proximal tubule cells (Tang et al., 1995). However, TGF-β_1_ seems to regulate Na,K-ATPase subunit expression in a cell type specific manner. Some studies showed that TGF-β_1_ reduced β_1_-subunit as well as α_1_-, α_2_-, and α_3_-subunit mRNA levels in young FRTL-5 rat thyroid cells (Pekary et al., 1997). Studies from our group found that although TGF-β_1_ decreased the surface levels of β_1_-subunit in kidney cells, this seemed to occur at the post-translational level since no significant changes in mRNA levels were observed between control and TGF-β_1_-treated cells (Rajasekaran et al., 2010). TGF-β_1_ also prevented the increase in α_1_-subunit mRNA induced by steroid hormones (Husted et al., 2000). Nevertheless, TGF-β_2_ selectively decreased the transcription of β_1_-subunit in ARPE-19 cells, a human retinal pigmented epithelial cell line, involving two transcription factors: hypoxia inducible factor (HIF) and Smad3 (Mony et al., 2013). HIF belongs to the basic helix-loop-helix (bHLH) superfamily and is a heterodimer composed of a hypoxia induced HIF-α subunit and a constitutively expressed HIF-1β subunit. Three HIF-α subunits have been identified so far: HIF-1α, HIF-2α, and HIF-3α. HIF-1α contains three major domains: a N-terminal domain, which is a DBD containing a bHLH; an oxygen-dependent degradation domain (ODDD) that mediates oxygen-regulated protein stability and a C-terminal domain, which recruits transcriptional coactivators such as CBP/p300 to activate gene transcription. The HIF complex associates with hypoxia response elements (HREs) in the regulatory regions of target genes and binds the transcriptional coactivators to induce gene expression. Besides hypoxia, HIF-1 is also regulated in an oxygen-independent manner (Ke and Costa, 2006). TGF-β_2_ increased HIF-1α expression in ARPE-19 cells and blocking the binding of HIF-1α to HREs with echinomycin prevented the TGF-β_2_-induced decrease of the β_1_-subunit (Mony et al., 2013). Since HIF-1α generally functions as a transcriptional activator, this observation raised the possibility that other transcription repressors may antagonize the function of HIF-1α in the regulation of Na,K-ATPase expression. The *ATP1B1* promoter region also contains a SBD in close proximity to a putative HRE. Smads are transcription modulators usually activated by TGF-β signaling that can inhibit gene expression by preventing the action of transcriptional activators (Massague et al., 2005). Studies in ARPE19 cells indeed support the idea that both Smad3 and HIF-1α cooperated in regulating the expression of β_1_-subunit expression by TGF-β_2_ (Mony et al., 2013).

### Fibroblast growth factor

Fibroblast growth factors (FGFs) control a wide range of biological functions and regulate cellular proliferation, survival, migration, and differentiation. FGFs are a family of glyocoproteins that are generally sequestered by the extracellular matrix and cell surface heparan sulfate proteoglycans. Activation of FGF signaling occurs upon release of the factor and binding to one of the four highly conserved FGF receptors, FGR1, FGR2, FGR3, and FGR4 (Turner and Grose, 2010). Incubation of cells in serum rapidly increased α_1_- and β_1_-subunit mRNA levels in VSMC and rat liver cells and induced *Atp1a1* and *Atp1b1* promoter activity, suggesting that serum regulates Na,K-ATPase gene expression at the transcriptional level (Kirtane et al., 1994; Nemoto et al., 1997). Serum differentially regulated α_1_- and β_1_-subunit mRNA induction and activation of PKC and tyrosine kinase activity was required for up-regulation of α_1_-subunit mRNA but not for β_1_-subunit. Further studies revealed that FGF treatment stimulated *Atp1a1* and *Atp1b1* promoter activities, similar to the observation in serum treated VSMC (Nemoto et al., 1997). Furthermore, keratinocyte growth factor (KGF), a member of the FGF family and a polypeptide mitogen secreted by fibroblasts and endothelial cells that acts primarily on epithelial cells, increased the abundance of α_1_-subunit, but not β_1_-subunit mRNA in alveolar type II cells (Borok et al., 1998).

## Prostaglandin and Na,K-ATPase transcription

Prostaglandin E1 (PGE1) is a member of the prostaglandin family, which are lipid mediators produced from arachidonic acid by cyclooxygenase and prostaglandin synthases. Prostaglandins exert their effects by activating G protein-coupled receptors and four PGE receptors have been reported: EP1 (E prostanoid receptor 1), EP2, EP3, and EP4 (Sugimoto and Narumiya, 2007). Prostaglandins are important regulators of ion transport in the kidney (Bonvalet et al., 1987) and PGE1 increased the transcription of α- and β-subunit of Na,K-ATPase in MDCK cells (Taub et al., 1992, 2004; Matlhagela and Taub, 2006). The increase in the transcription of β_1_-subunit induced by PGE1 was mediated by EP1 and EP2 receptors and PGE receptor antagonists inhibited the promoter activity of human *ATP1B1* induced by PGE1 (Matlhagela and Taub, 2006). Three prostaglandin response elements (PGRE) have been identified within the human *ATP1B1* promoter, which are located at position −445 to −438 bp (PGRE1: TGACCTTC); −226 to −216 bp (PGRE2: GTCCCTCA); and −92 to −100 bp (PGRE3: AGTCCCTGC; Matlhagela et al., 2005; Matlhagela and Taub, 2006) and PGRE1 is well conserved among species (Matlhagela and Taub, 2006). The PGRE sequence is similar to the consensus CRE, and CREB had been shown to bind to these sequences indicating that PGREs might be cAMP response elements. The binding affinity of CREB to three PGREs was varied with PGRE3 > PGRE1 > PGRE2 (Matlhagela et al., 2005). Sp1, Sp3 as well as CREB all bound to PGRE (Matlhagela and Taub, 2006) and the increase in transcription of β_1_-subunit was mediated through CREB binding to PGRE1 and PGRE3 as well as Sp1 binding to an adjacent Sp1 site (Matlhagela et al., 2005; Matlhagela and Taub, 2006). Mutations in PGRE3 or the GC box (−118 to −112 bp) immediately downstream of PGRE3 failed to mediate the increase in *ATP1B1* promoter activity induced by PGE1 (Matlhagela and Taub, 2006). Besides PGE1, PGE2 and PGF2α also have been shown to induce *ATP1B1* promoter activity in primary renal proximal tubule (RPT) cells (Herman et al., 2010).

## Na,K-ATPase activity and transcriptional regulation

Chronic inhibition of Na,K-ATPase activity by ouabain leads to an increase in the abundance of Na,K-ATPase. For example, ouabain increased the mRNA levels of α_1_- and β_1_-subunit in cultured rat astrocytes (Hosoi et al., 1997; Muto et al., 2000), which was abolished by actinomycin D, a transcription inhibitor acting by interfering with mRNA synthesis (Muto et al., 2000). Ouabain also regulated the transcription of α_3_- and β_1_-subunit in cultured neonatal rat cardiac myocytes. Ouabain augmented the *Atp1b1* promoter activity and the effect of ouabain on the regulation of Na,K-ATPase subunits was dependent on extracellular Ca^2+^ and calmodulin (Kometiani et al., 2000). The inhibition of Na,K-ATPase activity by ouabain can be mimicked by incubating cells in a low K^+^ environment and low extracellular K^+^ increased α_1_- and β_1_-subunit mRNA levels in rat cardiac myocytes (Qin et al., 1994; Zhuang et al., 2000; Wang et al., 2007a). Sp and CREB family transcription factors were required for the up-regulation of Na,K-ATPase subunits induced by low K^+^. Rat *Atp1a1* promoter region has a CRE/ATF site (at −70 to −63 bp) and a GC box motif (at −57 to −48 bp). Mutations in the CRE/ATF site or GC box substantially reduced low K^+^-mediated *Atp1a1* promoter activity. Low K^+^ increased the expression of Sp1, Sp3 and CREB-1 and enhanced the binding of these transcription factors to the GC box and, to a lesser extent, to the CRE/ATF site on rat *Atp1a1* promoter (Wang et al., 2007b). The sequences between -102 to +151 bp on rat *Atp1b1* were required for low K^+^-induced trans-activation of reporter gene expression (Qin et al., 1994). Low K^+^ enhanced the binding of Sp1 and Sp3 to GC box elements on the *Atp1b1* promoter (Zhuang et al., 2000). Mutations in potential GC box sequences decreased basal and low K^+^-mediated up-regulation of β_1_-subunit transcription in neonatal rat cardiac myocytes (Zhuang et al., 2000). Inhibiting the binding of Sp1 and Sp3 to GC boxes by mithramycin, which has high affinity for GC-rich DNA sequences (Matlhagela et al., 2005), blocked low K^+^-mediated up-regulation of *Atp1a1* and *Atp1b1* promoter activity. Multiple cellular signaling pathways were involved in this low K^+^-induced increase in the transcription of α_1_- and β_1_-subunits. Protein kinase A (PKA), ERK1/2, and histone deacetylase (HDAC) were required for up-regulation of α_1_-subunit transcription, whereas the transcription of β_1_-subunit was dependent on protein kinase C (PKC), c-Jun-N-terminal kinase (JNK) and p38 mitogen-activated protein kinase (MAPK; Wang et al., 2007a). However, it is important to point out that inhibition of Na,K-ATPase and therefore an increase in intracellular sodium regulates Na,K-ATPase on multiple levels including transcription, transport, endocytosis and degradation (Vinciguerra et al., 2003, 2004; Wang et al., 2014).

## Neuronal activity and Na,K-ATPase transcriptional regulation

α_1_-, α_3_-, and β_1_-subunits are abundantly expressed in most neurons in the central nervous system and neuronal activity modulates the transcription of Na,K-ATPase subunits. Depolarization by KCl increased the promoter activities as well as the mRNA levels of α_1_-, α_3_- and β_1_-subunits; however, impulse blockade induced by tetrodotoxin (TTX), a voltage-dependent Na^+^ channel blocker, significantly reduced α_1_-, α_3_-, and β_1_-subunit transcripts in murine neurons (Johar et al., 2012, 2014). At least two transcription factors were involved in this activity-dependent transcriptional regulation in neurons, nuclear respiratory factor 1 (NRF1) and the Sp family (Johar et al., 2012, 2014).

NRF1 is a transcription factor which was first identified as an activator of cytochrome C (Evans and Scarpulla, 1989). NRF1 increases the expression of nuclear genes required for mitochondrial biogenesis and function, but also binds to the promoter region of target genes which control cell cycle, cell growth, cell adhesion, migration, and tumor invasion (Okoh et al., 2011). Increased NRF1 levels have been observed in hepatoma, thyroid oncocytoma and breast cancers (Dong et al., 2002; Savagner et al., 2003; Okoh et al., 2011). The consensus NRF1 binding site is (T/C)GCGCA(C/T)GCGC(A/G), which is a GC-rich element. In addition, NRF1 binds to sequences with an invariant GCA core flanked by GC-rich regions, which can be found in 5′ flanking regions of *Atp1a1, Atp1a3*, and *Atp1b1* genes. It has been reported that NRF1 binds to the promoter regions of *Atp1a1* and *Atp1b1* in murine neurons at binding sites that are conserved among mice, rats and humans. Mutations in the NRF1 binding sites significantly decreased *Atp1b1* promoter activity, but increased *Atp1a1* promoter activity, which indicates that NRF1 differentially regulates Na,K-ATPase subunits. Indeed, silencing of NRF1 significantly decreased β_1_-subunit transcript but increased the mRNA level of α_1_-subunit and overexpression of NRF1 increased the mRNA level of the β_1_-subunit but decreased the α_1_-subunit transcript. NRF1 itself is regulated by neuronal activity (Yang et al., 2006). Depolarization by KCl increased NRF1 expression, and TTX reduced the NRF1 expression (Johar et al., 2012) and knockdown of NRF1 with small interference RNA blocked the up-regulation of the β_1_-subunit and down-regulation of the α_1_-subunit induced by KCl, whereas overexpression of NRF1 rescued the down-regulation of the β_1_-subunit and up-regulation of the α_1_-subunit by TTX. The inverse regulation of Na,K-ATPase α_1_- and β_1_-subunit by NRF1 in response to KCl and TTX points to additional transcription factors and an intricate network regulating Na,K-ATPase expression by neuronal activity.

Sp transcription factors are regulated by neuronal activity and depolarization by KCl increased Sp1 and Sp4 expression, while TTX reduced the expression of Sp1 and Sp4 (Johar et al., 2014). Sp1, Sp3, and Sp4 bound to the promoter regions of *Atp1a1, Atp1a3*, and *Atp1b1* in murine neurons, and Sp4 showed the highest binding affinity. Mutations in the Sp binding sites of *Atp1a1, Atp1a3*, and *Atp1b1* promoters significantly decreased the promoter activity. Silencing Sp1, Sp3, and Sp4 decreased α_1_-, α_3_-, and β_1_-subunit mRNA levels; while overexpression of Sp1, Sp3, and Sp4 increased α_1_-, α_3_-, and β_1_-subunit transcripts. Silencing Sp1 or Sp4 also blocked the KCl-induced up-regulation of Na,K-ATPase subunits and overexpression of Sp1, Sp3, or Sp4 rescued the down-regulation of Na,K-ATPase subunits by TTX (Johar et al., 2014).

## Epigenetic mechanisms in the regulation of Na,K-ATPase transcription

DNA methylation is an important epigenetic mechanism in the regulation of gene expression. Methylation generally occurs at the cytosine bases of CpG sequences throughout the entire genome, with the exception of CpG islands usually found in gene promoter regions (Cedar and Bergman, 2009). Methylation patterns often depend on tissue type and developmental stage, correlating with transcriptional activity. Aberrant DNA methylation patterns have been linked to altered gene expression in various genetic diseases and tumors (Bird, 2002). Higher methylated CpGs were found in the first exon of *Atp1a3*, which correlated with the lower expression of this gene in pig liver (Henriksen et al., 2013). Manganese exposure led to sustained *Atp1a3* promoter hypermethylation and downregulation of its transcript level in mice (Wang et al., 2013). CpG islands are also found in *ATP1B1* and *Atp1b2* promoter regions (Alvarez de la Rosa et al., 2002; Selvakumar et al., 2014), which are conserved between human and mice and remain unmethylated (Alvarez de la Rosa et al., 2002). However, *ATP1B1* promoter is hypermethylated in tumor samples of clear cell renal cell carcinoma, which showed reduced β_1_-subunit levels (Selvakumar et al., 2014). Knock-down of the tumor suppressor gene von Hippel-Lindau (VHL) enhanced *ATP1B1* promoter methylation and decreased β_1_-subunit expression, while inhibition of methyltransferase by 5-Aza-2′-deoxycytidine rescued β_1_-subunit mRNA expression in VHL-knockdown cells (Selvakumar et al., 2014).

Methylation is also involved in transcriptional regulation and differential distribution of FXYD1. The *FXYD1* promoter contains methylated cytosines and a predicted CpG island. 5′-aza-cytidine markedly increased *FXYD1* mRNA levels. Mouse heart showed lower methylation in *fxyd1* promoter and higher fxyd1 expression; while brain had a higher methylation of the *fxyd1* promoter and lower fxyd1 expression (Deng et al., 2007). In brain, the fxyd1 mRNA level is lower in the frontal cortex than in cerebellum, which correlates with the more frequent promoter methylation found in frontal cortex (Banine et al., 2011). The *fxyd1* promoter is a target of the Methyl CpG binding protein 2 (MeCP2), a common transcriptional modulator. MeCP2 directly bound to methylated CpG in the *fxyd1* promoter and repressed *fxyd1* transcription (Deng et al., 2007). On the other hand, activating histones, such as histone 3 acetylated at lysines 9 and 14 (H3K9/14ac) and histone 3 methylated at lysine 4 (H3K4me3), disassociated with the *fxyd1* promoter to augment the inhibitory effect (Banine et al., 2011).

## Conclusions

The expression of Na,K-ATPase is transcriptionally regulated by hormones, growth factors, lipid mediators and other extracellular stimuli through mediating transcription factor binding to promoter regions of Na,K-ATPase subunits. Many regulators of Na,K-ATPase are involved in the response to demands of cellular activity and ion homeostasis. Together with the spatial and temporal regulation of Na,K-ATPase subunits and their individual isoforms, this allows for a complex and controlled regulation of Na,K-ATPase in response to physiological demands in a tissue-specific manner. Interestingly, various recently identified transcriptional regulators of Na,K-ATPase are transcription factors that are also activated during development, in stem cells and during epithelial-mesenchymal transition (EMT). EMT is a switch in which epithelial cells undergo a shift from a well-differentiated polarized epithelial phenotype to a fibroblastic, mesenchymal phenotype and is a natural developmental process in tissue and organ formation. EMT can also be activated during wound healing, in tissue fibrosis, and is a major factor in cancer developing into a malignant disease (Kalluri and Weinberg, 2009). Vice versa, EMT activated transcription factors regulate the expression of Na,K-ATPase. For example, TGF-β and FGF up-regulate the expression of ZEB1, ZEB2, and Snail; steroid hormones, IGF-1 and PGE2 also induce ZEB1 and ZEB2. And ZEB1 and Snail1 regulate the transcription of Na,K-ATPase. Most recently, we identified the β_1_-subunit as a target of the Sonic hedgehog (Shh) signaling pathway (Lee et al., 2015). Shh is a critical morphogen involved in patterning of the early embryo and organogenesis, including the neural tube and limb system. The correlation between these signaling pathways and the transcriptional regulation of Na,K-ATPase is intriguing as elucidating the cross-regulation network among these factors may help in our understanding of the role of Na,K-ATPase with its pump-dependent and pump-independent functions in normal development, stem cell biology, and disease.

### Conflict of interest statement

The authors declare that the research was conducted in the absence of any commercial or financial relationships that could be construed as a potential conflict of interest.

## Abbreviations

CRE: cAMP response element
EMT: epithelial-mesenchymal transition
FGFs: fibroblast growth factors
GR: glucocorticoid receptor
GRE: glucocorticoid response element
HRE: hypoxia response element
MR: mineralocorticoid receptor
MRE: mineralocorticoid response element
Na,K-ATPase: sodium, potassium-adenosine triphosphatase
NRF1: nuclear respiratory factor 1
PGE1: prostaglandin E1
PGRE: prostaglandin response element
PR: progesterone receptor
PRE: progesterone response element
SBD: Smad binding domain
Sp: specificity protein
SRE: serum response element
TGF-β: transforming growth factor-β
TR: thyroid hormone receptor
TRE: thyroid hormone response element
ZEB1: zinc finger E-box binding homeobox.
